# Influence of smoking reduction on depressive and anxiety symptoms and quality of life in smokers with type 2 diabetes: a study focusing on the role of gender

**DOI:** 10.1192/j.eurpsy.2023.1223

**Published:** 2023-07-19

**Authors:** K. Tzartzas, A. Chappuis, C. Clair

**Affiliations:** 1 Département des Policliniques (DDP), Unisanté - Centre de médecine générale et de santé publique; 2 Unil, Lausanne, Switzerland

## Abstract

**Introduction:**

There is a complex relationship between smoking, mental health and diabetes. On one side, there is a bidirectional relationship between tobacco and depression: smokers are more at risk to suffer from depression compared with non-smokers and smoking cessation is associated with mood improvement. Moreover, persons with depressive disorders may be particularly vulnerable to nicotine addiction and are less likely to quit smoking. On the other side, a strong association is observed between depression and diabetes. Gender and sex have also been described as influencing the associations between these different pathologies.

**Objectives:**

Our aim was to determine if and how this relationship exist in a population with diabetes and to assess the impact of smoking reduction on depressive, anxiety symptoms and a health assessment score. We tried also to highlight the potential differences that we find between men and women.

**Methods:**

Data were collected from a randomized controlled trial evaluating the 1-year efficacy of smoking cessation interventions in a population of smokers with type 2 diabetes. PHQ-9, GAD-7 and SF12 scores were used to assess depressive, anxiety symptoms and health assessment. We used STATA to perform data analysis.

**Results:**

48 participants were recruited in this study. Women represent 60,4% of the population and men 39,6%. The mean age was 61,9 years old (SD 9.93). The mean depression, anxiety and health assessment scores were 4.1 (SD 4.55), 3.4 (SD 4.83) and 3.0 (SD 0.69) respectively. There were no significant differences between women and men scores. Women tended to have higher depression and anxiety scores, whether they reduced their consumption or not. Men who decreased their smoking consumption (reducers) tended to have better mental health scores than women at baseline. In both genders, we found a trend toward improvement in depressive, anxiety and health assessment scores of participants that reduced their tobacco consumption. We assessed a loss of 1.8 (SD 2.58, p-value= 0.424 ) and 1.7 (SD 3.20, p-value=0.448 ) points to their depressive and anxiety scores respectively while non-reducers only lost 0.6 (SD 4.03*) and 0.7 (SD 2.88**) points respectively.

**Conclusions:**

Our study suggests that differences between genders exist and that there are improvement differences in scores of people who reduced their tobacco consumption compared to people who didn’t reduce their consumption. However, none of our results were significant. A study with a greater number of participants and with more strength should be done to confirm our hypotheses.

**Disclosure of Interest:**

None Declared

